# Lipid, blood pressure and kidney update 2013

**DOI:** 10.1007/s11255-014-0657-6

**Published:** 2014-02-27

**Authors:** Maciej Banach, Corina Serban, Wilbert S. Aronow, Jacek Rysz, Simona Dragan, Edgar V. Lerma, Mugurel Apetrii, Adrian Covic

**Affiliations:** 1 Department of Hypertension, Chair of Nephrology and Hypertension, Medical University of Lodz, Zeromskiego 113, 90-549 Lodz, Poland; 2 Department of Functional Sciences, Chair of Pathophysiology, “Victor Babes” University of Medicine and Pharmacy of Timisoara, Timisoara, Romania; 3 Cardiology Division, New York Medical College, Valhalla, NY USA; 4 Department of Nephrology, Hypertension and Family Medicine, Chair of Nephrology and Hypertension, Medical University of Lodz, Lodz, Poland; 5 Cardiology Department, University of Medicine and Pharmacy “Victor Babes” Timisoara, Timisoara, Romania; 6 Section of Nephrology, University of Illinois at Chicago, Chicago, IL USA; 7 Nephrology Clinic, Dialysis and Renal Transplant Center, C.I. Parhon University Hospital, Iasi, Romania; 8 Grigore. T. Popa University of Medicine and Pharmacy, Iasi, Romania

**Keywords:** Anemia, Blood pressure, Cholesterol, Dyslipidemia, Hypertension, Lipids, Renal disease, Transplantation

## Abstract

The year 2013 proved to be very exciting as far as landmark trials and new guidelines in the field of lipid disorders, blood pressure and kidney diseases. Among these are the International Atherosclerosis Society Global Recommendations for the Management of Dyslipidemia, European Society of Cardiology (ESC)/European Society of Hypertension Guidelines for the Management of Arterial Hypertension, American Diabetes Association Clinical Practice Recommendations, the Kidney Disease: Improving Global Outcomes Clinical Practice Guidelines for Managing Dyslipidemias in Chronic Kidney Disease (CKD) Patients, the American College of Cardiology/American Heart Association Guideline on the Treatment of Blood Cholesterol to Reduce Atherosclerotic Cardiovascular Risk in Adults, the Joint National Committee Expert Panel (JNC 8) Evidence-Based Guideline for the Management of High Blood Pressure in Adults, the American Society of Hypertension/International Society of Hypertension Clinical Practice Guidelines for the Management of Hypertension in the Community, the American College of Physicians Clinical Practice Guideline on Screening, Monitoring, and Treatment of Stage 1–3 CKD and many important trials presented among others during the ESC Annual Congress in Amsterdam and the American Society of Nephrology Annual Meeting—Kidney Week in Atlanta, GA. The paper is an attempt to summarize the most important events and reports in the mentioned areas in the passing year.

## Lipid update 2013

### LDL cholesterol and coronary risk

In patients with multiple cardiovascular (CV) risk factors, it is essential to effectively manage the overall risk, in order to prevent CV events [1]. Traditionally, low-density lipoprotein cholesterol (LDL-C) and high-density lipoprotein cholesterol (HDL-C) have been considered as the classical biomarkers of risk assessment as well as the therapeutic targets in both primary and secondary prevention.

It is worth emphasizing that the current European Society of Cardiology (ESC)/European Atherosclerosis Society (EAS) guidelines (2011) indicate LDL-C as an only target for lipid disorders therapy [2]. Previous classification schemes and treatment levels for hyperlipidemia have been based on the National Cholesterol Education Panel’s Adult Treatment Program-3 (ATP-III) guidelines. Interestingly, in November 2013, the Kidney Disease: Improving Global Outcomes (KDIGO) published a new evidence-based Clinical Practice Guideline making recommendations on treatment of dyslipidemias in chronic kidney disease (CKD) [3]. One of the highlights of this was the recommendation against the use of LDL-C for assessing coronary risk in patients with CKD. The reviewed published evidence showed weak and potentially misleading association between LDL-C and coronary risk particularly in those with CKD, thereby mitigating against the use of LDL-C for identifying CKD patients who should receive lipid-lowering therapies. Nevertheless, the KDIGO Work Group recommended that follow-up measurement of lipid levels should be reserved for instances in which the results would alter management, e.g., assessment of adherence to statin treatment, change in renal replacement modality or concern about the presence of new secondary causes of dyslipidemia, or assessment of 10-year CV risk in patients younger than 50 years who are not currently receiving a statin [3, 4].

Later that month, the American College of Cardiology (ACC) and the American Heart Association (AHA) published very expected clinical practice guidelines for the treatment of cholesterol in those at high risk of atherosclerotic cardiovascular diseases (ASCVD) [5]. Corollary to the KDIGO guidelines [3, 4], the ACC/AHA recommendations did not focus on specific target levels of LDL-C and instead focused on four major groups of patients who are most likely to benefit from statin therapy, in terms of decreasing CV complications. These are: (1) patients with CVD, (2) patients with an LDL-C 190 mg/dL or higher, (3) patients with type 2 diabetes who are between 40 and 75 years of age and (4) patients with an estimated 10-year risk of CVD of 7.5 % or higher (based on new risk equation) who are between 40 and 75 years of age [5]. New risk assessment tools have also been recommended to complement the guidelines when embarking on the decision whether or not to start patients on statins [5].

The large debate has started since the publishing of the new lipid guidelines. In the same month, the National Lipid Association (NLA) released a position statement expressing opposition to the former’s recommendation to remove LDL-C (and non-HDL-C) treatment targets [6]. Also European Atherosclerotic Society (EAS) distanced from new ACC/AHA guidelines [7]. The European experts indicate that in the new American guidelines, statin treatment is recommended for primary prevention in subjects with a risk of ASCVD event of 7.5 %, irrespective of LDL-C level, which would correspond to a moderate—2.5 % risk of CVD death in 10 years according to the European SCORE model. Therefore, they suggest that the impact of the ACC/AHA strategy should be put into the perspective of the very large number of subjects in the population who would be eligible for lifelong statin treatment from the age of 40 years onwards [7]. They also comment a new risk estimation model for estimating the total CVD risk (Pool cohorts equations) that has been developed in the new guidelines and suggest that from the available documents it cannot be evaluated how this would work in relation to the European SCORE model. Therefore, they suggest that for the European population the SCORE charts or national charts calibrated on SCORE should be still recommended [7]. Finally, EAS guideline committee comments no treatment goals of LDL-C in new ACC/AHA guidelines, although the option of having treatment goals has been accepted. They indicate that treatment goals are widely used in different clinical settings, such as for the treatment of arterial hypertension or type-2 diabetes, and targets are a most important tool in daily practice, aiding patient-to-doctor communications and optimizing compliance, and emphasize that risk reduction in general should be individualized for each patient, and this can be more specific if targets are defined [7]. Finally, they take a notice that the EAS/European Society of Cardiology (ESC) (2011) guidelines have a broader approach on dyslipidemia in general, while the ACC/AHA guidelines are focused on statin treatment in cardiovascular prevention. Therefore, in the EAS/ESC guidelines, special groups, such as individuals with familial hypercholesterolemia, combined hyperlipidaemia and diabetes, and stroke patients, are discussed more in detail. What is also very important the EAS/ESC guidelines also include a more in-depth discussion and options on drug treatments other than statins, while in ACC/AHA lipid guidelines no other lipid-lowering drugs (as well as the combined therapy) is discussed and recommended [7]. It is also worth mentioning the other limitations of the American guidelines: (1) lack of inclusion of all important randomized controlled trials (RCTs) on statin therapy and therapeutic goals (the selectivity of RCTs inclusion); (2) lack of information on management with patient with side effects of statin therapy (including management in patients with statin intolerance); (3) the arbitrarily accepted age limit for the elderly patients (≥75) [8–11].

### New biomarkers of lipid disorders

Considering the complicated mechanisms and signals involved in atherosclerosis, research is now focused on novel lipid biomarkers that can be introduced as routine diagnostic tests. It is important to determine whether adding information on apolipoprotein B (apoB) and apolipoprotein A1 (apoA1), lipoprotein (a) or lipoprotein-associated phospholipase A2 to total cholesterol (TC), LDL-C and HDL-C improves cardiovascular disease (CVD) risk prediction [12–14]. It is known that in selected individuals at high CV risk, despite LDL-C, triglycerides (TG) should be targeted, but HDL-C, Lp(a) and ratios such as LDL-C/HDL-C or apoB/apoA1 are not recommended as treatment targets [15]. We still do not have enough data for these biomarkers (or the existing data suggest that we should not use the given biomarker as a treatment target), or their measurements are still too expensive and therefore not cost-effective (like for apoB).

Different clinical conditions associated with inflammation, oxidation, advanced glycation and protein carbamylation, such as diabetes, coronary artery disease (CAD) or CKD can alter the functionality of HDL, converting normal HDL into so-called dysfunctional HDL which is no longer cardioprotective [16]. Furthermore, it is widely accepted that the functionality of HDL subclasses defines the antiatherogenic quality of HDL [17]. The heterogeneity of HDL particles in terms of shape, size and apolipoprotein composition was shown to determine their ability to inhibit LDL oxidation and reduce migration of monocytes within the arterial wall [18, 19]. Dysfunctional HDL loses the function of reverse cholesterol transport and might exhibit pro-inflammatory, pro-oxidant, pro-thrombotic and pro-apoptotic properties, all responsible for the subsequent endothelial dysfunction [19, 20].

However, we still need a direct method to measure dysfunctional HDL, as currently we use many indirect methods, including the ones concerning the analysis of subfractions/subpopulations of lipoproteins [18, 19]. Recently, by means of one of these methods—an electrophoretic method (using LipoPrint system, Quantimetrix, USA), a new clinical phenomenon, atherogenic normolipidemia, has been described in healthy volunteers with no sign of overt CV disease [21, 22]. Despite normal levels of LDL-C, these subjects were still at a high CV risk due to high levels of sdLDL (LDL3–7 subfractions). So, both the “quality” and the “quantity” of plasma lipids and lipoproteins seem to essentially influence CV risk [23].

### Statin therapy update 2013

Despite well-established roles in primary and secondary prevention of CVD, due to their positive effects on the plasma lipid profile, statin use is associated with some side effects and residual risk [24]. Beyond their potent pharmacologic inhibition of cholesterol biosynthesis, statins appear to have pleiotropic effects, including antiarrhythmic, antiinflammatory, antioxidative, antithrombotic, antimitotic, antibacterial, C reactive protein-lowering, angiogenic, immunomodulatory and vascular protective (stabilization of the atheroma plaque) activity, inhibition of smooth muscle cell proliferation and migration, inhibition of cardiac remodeling, inhibition of matrix metalloproteinase and cyclooxygenase-2, inhibition of telomere shortening, and improvement of microvascular function (amelioration of endothelial function) and of autonomic nervous system function [25–27]. Through modulation of many known and unknown pathways, statins may influence a wide range of diseases such as heart failure, hypertension, atrial fibrillation, diabetes mellitus, CKD and cancer [28, 29].

#### Statins and new onset diabetes (NOD)

The first meta-analysis that revealed that statin therapy for a mean follow-up of 4 years was associated with a higher incidence (by 9 %) of NOD was published in 2010 [30]. Then, the next meta-analysis that included over 57,000 participants demonstrated an even higher, 13 % increase in the risk of NOD [31]. A careful review of findings from combined trials showed that statins can modestly raise blood glucose, and more patients who are on statin therapy are diagnosed with diabetes mellitus compared with those not on statins [32]. In February 2012, the Food and Drug Administration (FDA) released changes to statin safety label to include that statins have been associated with increases in hemoglobin A1C and fasting serum glucose levels. Furthermore, estimated risk of NOD from statin treatment is approximately one in 255–498 patients over 4 years [31–33]. At the same time, the number of patients needed to treat with high-dose statin therapy to prevent one CV event was 155 (2–3.5 times less than the risk of NOD) [31, 34]. There is still discussion on the possible mechanisms of pro-diabetic role of statins. Statins may affect molecular mechanisms that adversely impact on insulin sensitivity and beta-cell function, thereby increasing risk of NOD [35, 36]. Recently, a retrospective cohort study examined the incidence of NOD in primary care patients treated with statins also observed an increased risk of NOD in these patients [37]. A population-based case–control study in women from an Asian country found out that the risk of statin-related NOD was more evident in women aged 40–64 years compared with women aged 65 or more and was cumulative dose-dependent [38]. Another population-based study evaluated the risk of incident diabetes in more than 1.5 million older patients from Canada, treated with statins [39]. They found that, compared with pravastatin, treatment with atorvastatin, rosuvastatin or simvastatin, but not fluvastatin or lovastatin, was associated with an increased risk of incident diabetes in statin-naive older patients without diabetes [40]. On the contrary, another study that evaluated the risk of incident DM in relation to statin prescription in 4,750 hypertensive, non-diabetic outpatients showed that in real-life outpatient environment, statin prescription for primary prevention is not associated with increased risk of incident DM [41].

Because diabetes is a risk equivalent condition for vascular diseases, recent findings create a paradox whereby needed statin therapy may be withheld to avoid excess risk of diabetes, while representing the strongest cardiovascular risk reduction tool in diabetics [34, 41]. Therefore, the experts in the field of lipid disorders have recently recommended the statin-associated risk of NOD appears to be unrelated to specific statins, but it seems to be dose-dependent [34]. They also indicate some risk factors increasing the risk of NOD in patients treated with statins. Changes in the LDL-C concentration do not influence the risk of NOD, but older age, higher baseline fasting glucose levels and other features of metabolic syndrome [42] are the strongest predictors of NOD [34]. Some studies also suggest that women, the elderly and the Asians are at particularly higher risk of NOD [34, 36]. There is also no doubt that statins should be used in secondary prevention patients, as the CV benefits significantly outweigh the risk of NOD [34, 36]. However, it is still questionable whether statins should be used for primary prevention among patients with a relatively low baseline CV risk (and with risk factors for diabetes). In these patients, it is recommended to use all possibilities of non-pharmacological therapy, and introducing statins should be considered individually after careful estimation of CV risk and treatment adverse events risks, when non-pharmacological therapy is not effective [34, 36, 43].

#### Statin use in hypertensive patients

Hypertension and hyperlipidemia often coexist and seem to be interrelated through common pathophysiological pathways [44]. The role of statins in controlling blood pressure (BP) in patients with hypertension has been controversial. There are several mechanisms by which statins could influence and modify BP values: increasing the production of nitric oxide (NO), inhibition of the production of reactive oxygen species (ROS), reducing large artery stiffness and improving systemic arterial compliance [45–50]. Until now, some studies indicated the possibility of BP lowering with statins, whereas others did not [47, 51]. On the basis of data from the available studies, it appears consistent that statins are useful in hypertensive patients irrespective of lipid profile, especially in patients with concomitant risk factors or CAD, as they significantly decrease the risk of all major cardiovascular outcomes (especially in secondary prevention, without the influence on all-cause mortality in the primary prevention) [47, 52]. The recent meta-analysis of randomized controlled trials that studied the effects of statins on blood pressure in normotensive or hypertensive subjects provided reliable evidence against any substantial BP-lowering effect of statins in both normotensive and hypertensive patients, suggesting that the established protective effects of these drugs on the CV system do not materially depend on reductions in BP [53, 54].

#### Statin use in chronic kidney disease patients

CKD is associated with CVD even in the early stages, and a large number of patients die before developing advanced CKD [26, 55–58]. The available data suggest that efforts to reduce mortality in the CKD population should be focused on treatment and prevention of, among others, CAD and congestive heart failure [58–61]. In the ESC/EAS 2011 guidelines, it is clearly stated that CKD patients should be automatically treated as subjects at very high or high total cardiovascular risk who need active management of all risk factors [2]. A Lipid and Blood Pressure Meta-analysis Collaboration (LBPMC) Group meta-analysis of randomized controlled trials showed that statin therapy significantly modifies the lipid profile in CKD patients not on dialysis therapy (with the trend to be more effective with longer therapy) and have less beneficial effect in patients on dialysis with the trend to be less effective with longer duration of therapy (and even with some harmful effects such as TG increase and HDL-C reduction) [62]. Another meta-analysis from the same group that included 6,452 CKD subjects randomized to receive either statin or placebo studied the role of statins on renal outcomes [63]. It was observed that statins might exert significant renoprotective effects in CKD patients depending on the duration of treatment (especially on urinary protein, serum creatinine and glomerular filtration rate up to 3 years), but only in patients without dialysis therapy [63]. Another meta-analysis of 11 randomized controlled trials involving 21,295 participants showed that statins decrease all-cause mortality only in CKD patients not requiring dialysis therapy [64].

The very recent position paper of International Atherosclerosis Society (IAS)—Global Recommendations for the Management of Dyslipidemia suggested that in CKD patients classified as moderately high risk, the optimal range of LDL-C should be <100 mg/dL (2.6 mmol/L) [65]. The recently published attempt at recommendation on statin use in patients with CKD suggests that CKD patients not requiring dialysis should be treated with statins for high CV risk and that the duration of treatment is particularly important for optimization of its effects [66]. It is also suggested that on the basis of available data, we cannot recommend initiating statin treatment in CKD patients requiring dialysis. However, on the other hand, we do not have enough data to stop treatment in patients who are already on statins [66]. This is the same stance that the KDIGO Work Group has taken as far as which particular patient population should receive statins [3, 4]. In contrast, however, and as previously noted, KDIGO recommended against the use of LDL-C for identifying CKD patients who should receive statins and also recommended that it is unnecessary to measure LDL-C in situations in which the results would not alter management decisions, e.g., those already receiving a statin (or in whom statin treatment is clearly indicated or not indicated based on changes in their cardiovascular risk profile or clinical status) would not require follow-up measurements of LDL-C [3, 4].

#### PCSK9 inhibitors

Insights from randomized controlled trials in patients with heart failure, atrial fibrillation and CKD suggest that there are still some questions regarding the role of statins in these conditions [67]. Statins activate LDL receptor (LDLR) gene expression, but also activate the expression of proprotein convertase subtilisin/kexin type 9 (PCSK9), a secreted inhibitor of LDLR, thereby limiting their beneficial effects [68]. PCSK9 is a serine protease expressed predominantly in the liver, intestine and kidney [69]. PCSK9 directly binds to the epidermal growth factor-like repeat A domain of the LDL receptor and induces its degradation, thereby controlling circulating LDL-C concentration [70, 71]. Recently, PCSK9 inhibition seems to be an attractive as a new strategy for lowering LDL-C levels, especially in combination with lipid-lowering drugs such as statins [72]. A new study that highlights differences in PCSK9 variants among Caucasian and African Canadians showed the PCSK9 gene to be highly polymorphic, with more than 50 exonic variations documented to have opposing effects on LDLC levels [73].

PCSK9 is able to induce degradation of the LDLR-related protein 1 (LRP-1), and although the latter is not an essential factor for LDLR regulation, the LDLR effectively competes with LRP-1 for PCSK9 activity. Identification of PCSK9 targets should allow a better understanding of the consequences of PCSK9 inhibition for lowering LDL-C [74]. Inhibition of the interaction between PCSK9 and the LDLR with monoclonal antibodies (mAbs) targeting PCSK9 has a great potential for patients with hypercholesterolemia (from the high risk groups), including familial hypercholesterolemia, as well as in patients with statin intolerance. Early clinical phase studies suggest that PSCK9 inhibitors given subcutaneously two or four times a month (both in monotherapy and in the combination with statin) are very effective reducing the baseline LDL-C even by 75 % and well tolerated [75–77]. However, further studies with longer follow-up (current observations were usually up to 12 weeks) with the analysis of PCSK9-inhibitors effect on primary and secondary endpoints (CV and mortality outcomes) are required to finally assess their efficacy and safety profile of this drugs [75, 78].

## Blood pressure update 2013

The American College of Cardiology Foundation (ACCF)/American Heart Association (AHA) 2011 expert consensus document on hypertension in the elderly developed in collaboration with the American Academy of Neurology, American Geriatrics Society, American Society for Preventive Cardiology, American Society of Hypertension, American Society of Nephrology, Association of Black Cardiologists and European Society of Hypertension recommended that the BP should be lowered to less than 140/90 mmHg in adults with hypertension younger than 80 years at high risk of CV events [79]. On the basis of data from the Hypertension in the Very Elderly Trial (HYVET) [80], these guidelines recommended that for those who 80 years of age and older, the systolic BP should be reduced to 140–145 mmHg if tolerated [79]. On the other hand, data from the subanalyses and other observational studies suggest that there might be some benefit in reducing systolic BP below 140 mmHg (see also below) [81–88]. The choice of specific antihypertensive agents depends on efficacy, tolerability, presence of specific comorbidities and cost [79].

Dyslipidemia often coexists with hypertension, and statins should be always considered in hypertensive patients, especially with other CV risk factors and CAD (see above) [47, 89–92]. Control of BP and serum LDL-C may significantly reduce progression of angiographic CAD [13].

In 2013, there have been several studies searching for new biomarkers that correlate with hypertension complications, such as cardiotrophin 1 (CT-1) and procollagen III N-terminal propeptide, which are early markers of left ventricular injury, as well as neutrophil gelatinase-associated lipocalin, which could be a sensitive marker of kidney function in elderly patients with hypertension [93, 94]. In many interesting papers published last year, the authors not only evaluated the biomarkers, but also analyzed the type of hypertension. It was, among other, showed that a non-dipping BP pattern might be responsible for development of left ventricular hypertrophy in patients with hypertension [95].

There have been also studies looking at improved control of hypertension, which remains as a large problem in both Europe and the US [96–98]. The Kaiser Permanente Northern California Registry included 652,763 patients with hypertension [99]. Use of a hypertension program improved control of hypertension from 43.6 to 80.4 % (*p* < 0.001 for trend) [99]. The prevalence of hospitalization attributable to hypertensive diseases among United States adults aged 35 and older increased in men and in women from 1980 to 2007 (*p* < 0.001), especially in the Southern region of the United States [100].

In recent years, there is also a large discussion on cardiometabolic risk at children. It has been recently showed that persons with persistently increased BP from childhood to adulthood had significantly increased risk of carotid atherosclerosis [101]. This risk was decreased if increased BP during childhood resolved by adulthood [101].

2013 is a year with new data on brain (cerebrovascular) damage in hypertensive patients. 3,020 patients (mean age 63 years) with a recent lacunar stroke were randomized in an open-label trial to a systolic blood pressure of 130–149 mmHg or of <130 mmHg; patients with a systolic blood pressure of 127 mmHg after 1 year had an insignificant 19 % reduction in all-stroke compared WITH patients with a systolic blood pressure of 138 mmHg after 1 year [102]. Further insights into this important issue will be provided by a new trial—Optimal Blood Pressure and Cholesterol Targets for Preventing Recurrent Stroke in Hypertensives (ESH-CHL-SHOT)—which starts recruiting patients this year [103]. But, on the basis of available trials, it seems that there is a linear relation between stroke outcomes and systolic blood pressure, without any J-curve relation [104, 105].

There were also some new guidelines published in 2013. The American Diabetes Association (ADA) 2013 guidelines recommend that diabetics with hypertension should have their systolic blood pressure reduced to less than 140 mmHg [106]. A systolic blood pressure less than 130 mmHg may be considered in younger patients with long life expectancy if achieved with few drugs and without side effects [106]. The drug regimen should include an angiotensin-converting enzyme inhibitor or angiotensin receptor blocker unless the patient is pregnant. In pregnant women with chronic hypertension, a suggested target blood pressure is 110–129/65–79 mmHg [106]. The KDIGO guidelines for management of BP in patients with non-dialysis-dependent CKD published in December 2012 recommended that adults with CKD without diabetes mellitus [107] or with diabetes mellitus [108] with hypertension and albuminuria less than 30 mg per 24 h should have their BP reduced to ≤140/≤90 mmHg with a class IB indication. If albuminuria greater than 30 mg per 24 h is present in these patients, reduction in the BP to ≤130/≤80 mmHg has a class IID indication which we would not follow [107, 108]. The European Society of Hypertension (ESH)/European Society of Cardiology (ESC) 2013 guidelines for the management of hypertension [109] recommend reducing the systolic blood pressure to less than 140 mmHg in all patients at low to moderate cardiovascular risk (class I indication), in patients with diabetes mellitus (class I indication), in patients with a prior stroke or transient ischemic attack (class IIa indication), in patients with coronary heart disease (class IIa indication) and in patients with diabetic or non-diabetic CKD (class IIa indication) [109]. In elderly patients younger than 80 years with a systolic blood pressure of 160 mmHg or higher, the systolic blood pressure should be reduced to between 140 and 150 mmHg (class I indication) with consideration of a systolic blood pressure less than 140 mmHg (class IIb indication) [109]. In patients older than 80 years with a systolic blood pressure of 160 mmHg or higher, the systolic blood pressure should be reduced to between 140 and 150 mmHg provided they are in good physical and mental conditions (class I indication). A diastolic blood pressure target of less than 90 mmHg is always recommended except in diabetics in whom a level less than 85 mmHg is recommended (class I indication) [109]. These guidelines also recommend in resistant hypertensive patients withdrawing drugs if their antihypertensive effect is absent or minimal (class I indication), consider adding a mineralocorticoid receptor antagonist, amiloride or doxazosin if no contraindication exists (class IIa indication), and consider in truly drug resistant hypertension with BP confirmed by ambulatory blood pressure monitoring an invasive procedure such as renal denervation or baroreceptor stimulation (class IIb indication) [109].

However, it is worth emphasizing that we have still had very limited data on optimal BP levels in the elderly [110–113]. The REasons for Geographic and Racial Differences in Stroke (REGARDS) study is an observational study of risk factors for stroke [114]. This study included 4,181 persons aged 55–64 years, 3,767 persons aged 65–74 years, and 1,839 persons aged 75 years and older (mean 79.3 years). Median follow-up was 4.5 years for CVD (first occurrence of a coronary heart disease or stroke event), 4.5 years for CHD (non-fatal myocardial infarction or coronary heart disease death), 5.7 years for stroke and 6.0 years for all-cause mortality. The results from this study generated a hypothesis that for all patients older than 55 years, the recommended level of systolic blood pressure should be less than 140 mmHg with optimal values possibly between 120 and 139 mmHg [114].

In December 2013, the Joint National Committee Expert Panel (JNC 8) published their Evidence-Based Guideline for the Management of High Blood Pressure in Adults [115]. The most important changes from the preceding JNC 7 guidelines included the following:

- In patients 60 years of age or older who do not have diabetes or CKD, the goal blood pressure level is <150/90 mmHg, whereas in patients 18–59 years of age without major comorbidities, and in patients 60 years of age or older who have diabetes, CKD or both, the goal blood pressure is <140/90 mmHg. In younger patients without major comorbidities, increased DBP is a more important CV risk factor than is increased SBP, whereas in patients 60 years of age and older SBP control remains the most important factor (as in JNC 7) [115];
- First-line and later-line treatments are now be limited to four classes of medications namely: thiazide-type diuretics, calcium channel blockers (CCBs), angiotensin-converting enzyme inhibitors (ACE-I) and angiotensin II receptor blockers (ARB). Second- and third-line agents include higher doses or combinations of ACE-I, ARB, thiazides and CCBs [115];
- For black patients without CKD, initial choices of antihypertensives should include CCBs and thiazides instead of ACE-I. Use of ACE-I and ARB is recommended for all patients with CKD regardless of ethnic background, either as first-line therapy or in addition to first-line therapy [115];
- ACE-I and ARB should not be used in the same patient simultaneously. This has been supported by three trials, namely the ONgoing Telmisartan Alone and in combination with Ramipril Global Endpoint Trial (ONTARGET) [116], the Aliskiren Trial in Type 2 Diabetes Using Cardio-Renal Endpoints (ALTITUDE) [117] and the recently published the Veterans Affairs Nephropathy in Diabetes (VA-NEPHRON) [118] which showed that hyperkalemia was significantly higher with combination therapy than with monotherapy (6.3 vs. 2.6 events per 100 person-years; *p* < 0.001), as was acute kidney injury (12.2 vs. 6.7 events per 100 person-years; *p* < 0.001). At a median follow-up of 2.2 years, there was no significant difference in the study’s primary endpoint of renal disease progression or death between the monotherapy and combination therapy groups (152 vs. 132; *p* = 0.30). There was also no difference in cardiovascular events [115, 118].
- CCBs and thiazides should be used instead of ACE-I and ARB in patients over the age of 75 with impaired kidney function due to the risk of hyperkalemia, increased creatinine and further renal impairment [115].

Later, the American Society of Hypertension (ASH) and the International Society of Hypertension (ISH) issued separate guidelines from JNC 8 [119], with some important differences:

- They recommended that the start treatment threshold of >150/90 mmHg applies to patients 80 years or older.
- They also suggested different antihypertensives for initial therapy, based on the patients’ race, age and blood pressure level. They recommended an ACE-I or ARB for non-black patients under age 60 years of age, and a CCB or thiazide-type diuretic for non-black patients over 60 years of age. For black patients, they recommended a CCB or thiazide-type diuretic. In patients with blood pressure of at least 160/100, they recommended starting with two drugs from the start and they also had separate drug recommendations for special populations.

Several noteworthy trials on hypertension also deserve mention at this point. Over the past several years, renal angioplasty and stenting of atherosclerotic renal artery stenosis (RAS) became a common procedure. Recently, however, two randomized trials; the ASTRAL [120] and STAR [121] trials have failed to show any benefit. In December 2013, the results of CORAL trial [122] were published and demonstrated no difference in the primary and composite endpoints of death, myocardial infarctions, stroke, heart failure, progression of CKD and need for RRT. The only exception was for that of BP in which a significant but minor (2 mmHg) drop in the intervention arm [122]. Therefore, for the majority of patients with RAS and either hypertension or CKD, management of RAS should be limited to medical therapy. Nevertheless, it remains to be seen if certain patients might still obtain some benefit from this procedure, e.g., those with severe stenosis to a single functioning kidney or those with severe stenosis and AKI and those presenting with flash pulmonary edema [120–122].

With the burden of resistant hypertension, the concept of renal denervation has been introduced. This is based on the premise that in patients with resistant hypertension, there is overstimulation of the sympathetic nervous system, i.e., afferent signaling from the kidneys increases central sympathetic drive, while efferent signals to the kidneys increase renin release and sodium retention, while reducing renal blood flow. Catheter-based renal denervation cuts this communication between the kidneys and the sympathetic nervous system, and theoretically will result in lowering of blood pressure [123, 124]. The Catheter-Based Renal Sympathetic Denervation for Resistant Hypertension: A Multicenter Safety and Proof-of-Principle Cohort Study (SYMPLICITY HTN-1) [123] involving 45 patients was published in 2009, followed by the renal sympathetic denervation in patients with treatment-resistant hypertension trial (SYMPLICITY HTN-2 in 2010) [124], involving 106 patients, and both studies showed a significant decrease in BP in a select group of patients with resistant hypertension subjected to catheter-based renal denervation. However, the pivotal the Renal Denervation in Patients With Uncontrolled Hypertension trial (SYMPLICITY-3) [125] (initiated recruitment in 2011) which randomized 535 patients was abruptly discontinued in January 2014, as it allegedly failed to show that treatment with the novel procedure resulted in a sustained reduction in systolic blood pressure [125]. According to the company, no safety (the primary safety endpoint was the incidence of major adverse events that occurred one month after treatment until six months) issues arose during the study [126].

## Kidney update 2013

### Screening and kidney disease

In October 2013, the American College of Physicians (ACP) published guidelines [127] to present the evidence and provide clinical recommendations on the screening, monitoring and treatment of adults with stage 1–3 CKD. This included four recommendations, which are the following:

- ACP recommended against screening for CKD in asymptomatic adults without risk factors for CKD (grade: weak recommendation, low-quality evidence);
- ACP recommended against testing for proteinuria in adults with or without diabetes who are currently taking an ACE-I or ARB (grade: weak recommendation, low-quality evidence);
- ACP recommended that clinicians select pharmacologic therapy that includes either an ACE-I (moderate-quality evidence) or an ARB (high-quality evidence) in patients with hypertension and stage 1–3 CKD (grade: strong recommendation);
- ACP recommended that clinicians choose statin therapy to manage increased low-density lipoprotein in patients with stage 1–3 CKD (grade: strong recommendation, moderate-quality evidence) [127].

While the American Society of Nephrology (ASN) agreed with most of the above recommendations from ACP as they did reflect current standard clinical practice, they expressed disagreement with the ACP’s recommendation against screening for CKD in asymptomatic adults without risk factors as well as the recommendation not to test for proteinuria in adults with or without diabetes who were already being treated with either an ACE-I or ARB [128]. ASN President Bruce Molitoris opined that “If detected early in its progression, kidney disease can be slowed and the transition to dialysis delayed. This evidence-based fact is why regular screening and early intervention by a nephrologist is so important to stemming the epidemic of kidney disease in the United States and why ASN strongly recommends it.” This remains a controversial topic for debate in the years to come [128].

### Bone and the kidney

A tendency toward phosphate retention begins early in renal disease, due to the reduction in the filtered phosphate load. Phosphate retention is intimately related to the common development of CVD risk in CKD, increased fibroblast growth factor (FGF)-23 levels and secondary hyperparathyroidism [129]. The Kidney Disease Improving Global Outcomes (KDIGO) guidelines on CKD-Mineral and Bone Disorder diagnostics and treatment were published in 2009 and emphasize the need of phosphate measurements during the course of disease [130]. Phosphate binders in use for treatment of chronic hyperphosphatemia are generally categorized as calcium-containing (mostly calcium carbonate and calcium acetate) and non-calcium-containing (including sevelamer and lanthanum). As noted in the KDIGO guideline, all are effective in lowering phosphate. There is no consensus about whether any particular phosphate binder should be used in patients with CKD.

However, clinicians are becoming more cautious with the use of calcium-containing phosphate binders because of concerns about toxicity of calcium accumulation associated with vascular calcification or adynamic bone disease. The decision to use non-calcium-containing binders over calcium-containing binders was for many years a subject of debate. In a recent meta-analysis of 11 open-label trials (4,622 patients) revealed a 22 percent decrease in all-cause mortality among patients with CKD randomly assigned to receive non-calcium-based phosphate binders [131]. These results are of particular interests because there aren’t many strategies to improve mortality in CKD patients.

Given that the current approach to management of hyperphosphatemia is not always optimal, a number of alternative therapies are undergoing evaluation. These include colestilan and iron-containing phosphate binders. Colestilan is an anion exchanger compound with some similarities to sevelamer, initially being approved for the treatment of hypercholesterolemia. Recently, it has been successfully used as a phosphate binder for patients on dialysis in a phase III study, with similar effects compared with sevelamer or calcium-containing phosphate binders [132] in connection with significant lipid-lowering effects. However, data regarding mortality or other hard endpoints related to colestilan treatment in CKD patients are lacking.

Iron-containing phosphate binders represent other new alternatives in the treatment of hyperphosphatemia. Two molecules are most promising: iron(III)-oxyhydroxide-based phosphate binder (PA21) and ferric citrate, both being the subject of ongoing phase III studies. Even if appears that both products have similar efficiency with other phosphate binders with a lower pill burden, side effect profile and cost effectiveness remain to be evaluated [133, 134].^1^^,^ 2

### Acute kidney injury

The administration of radiocontrast media can lead to a usually reversible form of acute kidney injury (AKI) that begins soon after the contrast is administered [135]. Since there is no specific treatment once contrast-induced acute kidney injury (AKI) develops, the best treatment for contrast-induced kidney injury remains prevention. Traditionally, acetylcysteine or volume expansion using saline or sodium bicarbonate was the only methods proved to prevent contrast nephropathy. However, a variety of other interventions have been tried, including remote ischemic preconditioning. The latter is a method by which the deliberate induction of transient non-lethal ischemia of an organ protects against subsequent ischemic injury of another organ. The pathophysiological mechanism of remote ischemic preconditioning relies on a common final signaling pathway on the mitochondria. The mechanisms by which this occurs have been extensively investigated and involve signalling pathways in the mitochondria that prevent cell death [136]. Conditioning is currently being investigated in a number of clinical scenarios including renal protection.

An earlier randomized trial demonstrated that transient arm ischemia induced by intermittent blood pressure cuff inflation prior to contrast administration conferred protection against contrast nephropathy [137]. A second randomized trial in over 200 patients with a non-ST-segment increase myocardial infarction found that remote ischemic preconditioning induced by intermittent balloon catheter inflation at the time of percutaneous coronary intervention resulted in a lower rate of acute kidney injury [138].

#### Mortality risk with hydroxyethyl starch solution (HES)

Administration of intravenous fluids for volume expansion is very common in critically ill patients, particularly in the early stages of sepsis or during the perioperative period. A recent report of US Food and Drug Administration (FDA) indicates a higher risk of renal injury and mortality in critically ill patients treated with HES. The report recommends not using HES therapy in patients with preexisting renal dysfunction and to stop HES therapy at first sign of renal failure or coagulopathy. As HES fluids have higher associated costs than crystalloids, it seems reasonable to conclude that such fluids should not be used in critically ill patients [139].

#### Novel agent for treatment of hyperkalemia

A recent paper [140] questioned the rationale on the time-honored use of sodium polystyrene sulfonate (SPS), in the treatment of hyperkalemia, especially in light of the FDA warning issued in 2009, alluding to reports of colonic necrosis secondary to concomitant administration with sorbitol. The investigators found neither convincing evidence that SPS increased fecal potassium losses nor evidence that adding sorbitol increased its effectiveness. In November 2013, during the ASN Kidney Week, the company ZS Pharma presented the results of a phase II clinical trial of a novel potassium binder called ZS-9 (zirconium silicate), which was shown to be potentially capable of lowering potassium with minimal side effects [141, 142]. Now in phase III, ZS-9 is a highly selective oral sorbent (inorganic crystal) that selectively traps potassium ions over other ions throughout the gut. Interestingly, in in vitro studies, it was demonstrated to have a binding capacity of up to 3.5 mEq of potassium per gram. Its selectivity and unique structure allow it to be potentially effective and safe in rapidly removing excess potassium and maintaining normal levels with a relatively low drug burden [141, 142].

### Chronic kidney disease

#### New anemia drugs in CKD

Peginesatide is a synthetic peptide, attached to polyethylene glycol (“PEGylated”) [143] which mimics the structure of erythropoietin. It was approved in 2012 by the U.S. Food and Drug Administration for treatment of anemia associated with CKD in adult patients on dialysis [144]. The use of peginesatide was supported by two randomized studies: the EMERALD study in which peginesatide was not inferior to epoetin for patients receiving dialysis and the PEARL study in which peginesatide administered once monthly was compared with darbopoetin twice monthly with no differences regarding efficiency in non-dialyzed patients [145]. However, cardiovascular events and mortality were increased with peginesatide in patients with CKD who were not undergoing dialysis [146]. Furthermore, roughly 0.2 % of the 25,000 patients receiving the drug since its approval have experienced hypersensitivity reactions. Therefore, peginesatide has been voluntarily recalled by the manufacturers after these reports of anaphylaxis leading to three deaths.

#### Tolvaptan and liver damage

Tolvaptan is a selective, competitive vasopressin receptor 2 antagonist used to treat hyponatremia associated with congestive heart failure, cirrhosis and the syndrome of inappropriate antidiuretic hormone (SIADH). It was also demonstrated that it could be beneficial in the treatment for polycystic kidney disease by decreasing cAMP levels, inhibit cystogenesis [147]. According to a recent FDA alert, tolvaptan should not be used for more than 30 days or in patients with underlying liver disease. These patients are at increased risk of severe liver injury (potentially requiring liver transplantation) or death, according to the report.

#### Dialysis in the elderly

In our days, dialysis is widely available determining the nephrologists to consider its application in every patient in whom it might be indicated. Furthermore, the proportion of aged patients entering dialysis is fasting increasing over time. Overall, the initiating of dialysis in patients older than 70 years is associated with a better outcome as noted in a recent large retrospective study. However, the survival benefit was not observed among patients older than 80 years or among those with significant comorbidities. In addition, dialyzed patients were more prone to be hospitalized compared with patients on conservative care, which could have a negative impact on quality of life [148].

### Transplantation

#### Immunosuppression and risk of polyomavirus BK nephropathy

The human polyomaviruses BK are highly prevalent in humans but appear to cause clinical disease only in immunocompromised patients. BK virus primarily causes tubulointerstitial nephritis and ureteral stenosis in renal transplant with a medium reported prevalence of 5 % [149]. Hirsch and colleagues recently investigated the incidence of BKV replication in more than 600 de novo kidney transplant recipients who were randomly assigned to receive either tacrolimus or cyclosporin A. All patients received basiliximab induction therapy as well as mycophenolic acid and prednisone. According to the authors, the risk of polyomavirus BK viremia (BKV) in kidney transplant recipients was increased by high steroid exposure early after transplantation, treatment with tacrolimus rather than cyclosporin A, older donor age and male gender [150]. These results could be the result of a pharmacological interaction between steroid and tacrolimus, which might occur as a result of steroid-induced activation of cytochrome P450 3A and/or P-glycoprotein—enzymes that have a role in tacrolimus metabolism. Furthermore, cyclosporin A has been shown to suppress BKV replication in vitro but whether such effect is cell-specific and/or sufficient to offset the immunosuppressive properties of the agent in vivo is not known [151].

#### New guidelines on the management of CMV

Cytomegalovirus (CMV) remains one of the most common complications affecting organ transplant recipients, with significant morbidity, graft loss and occasional mortality [152]. The Transplantation Society International Cytomegalovirus (CMV) Consensus Group has published in 2013 new consensus guidelines on the management of CMV in solid-organ transplantation [153]. The guidelines discuss the immunology, prevention, treatment, drug resistance and pediatric-specific issues associated with CMV infection. These are the most important recommendations:

- Quantitative nucleic acid amplification testing (QNAT) is preferred for diagnosis, decisions regarding preemptive therapy and monitoring response to therapy due to the ability to harmonize and standardize these tests (strong, moderate). If QNAT is not available, antigenemia is an acceptable alternative; viral culture of blood or urine has a very limited role for the diagnosis of disease.
- Valganciclovir is increasingly used as the preferred agent for treatment. Additional specific recommendations on the use of IVIG with CMV treatment are included.
- Diagnostic resistance mutations have been updated, and the clinical management algorithm for ganciclovir-resistant CMV has been slightly modified to clarify decision-making criteria.
- In the pediatrics section, valganciclovir is included in the prevention and treatment of CMV due to new data detailing the pharmacokinetics of valganciclovir in pediatrics [153].

## Footnotes

1 First phase II study report on polynuclear iron(III)-oxyhydroxide phosphate binder (PA21) in hyperphosphatemic hemodialysis patients.

2 Prospective, phase III, multicenter, open-label, randomized clinical trial using ferric citrate in hyperphosphatemic hemodialysis patients.
